# Mammary hidradenitis suppurativa: case series, discussion of differential diagnoses and expanded classification^؆^

**DOI:** 10.1016/j.abd.2026.501410

**Published:** 2026-06-27

**Authors:** Ariany T.A.S. Denofre, Carolina M. Stecca, Thais dos S. Martins, Luciana V. Gomide, Juliana Y. Massuda Serrano, Thais H. Buffo, Renata F. Magalhães

**Affiliations:** Discipline of Dermatology, Universidade Estadual de Campinas, Campinas, SP, Brazil

Dear Editor,

Hidradenitis suppurativa (HS) affects women's quality of life, causing anatomical and functional damage to the breast region. Although four subtypes of breast involvement were proposed in 2021 (nodular, sternal, frictional, and mixed),^1^ these do not address all presentations (such as axillomammary or areolar lesions) nor describe the location of the nodular form. Surgical treatment is not standardized, resulting in deforming surgeries or refusal of intervention by surgeons for fear of recurrence.^2^ The main differential diagnoses, such as idiopathic granulomatous mastitis and squamous metaplasia, which cause fistulas/drainage similar to HS, should be discussed in more clinical, imaging, and therapeutic details.^3^, ^4^ The aim of this study is to evaluate the frequency and characteristics of breast involvement in HS, propose a classification expansion, and discuss differential diagnoses with ultrasound support for better clinical and surgical therapeutic management.

A retrospective study of 108 patients (68 women and 40 men) with HS followed at the outpatient clinic of Hospital das Clínicas of Unicamp from 2018 to 2022 was conducted. Patients over 18 years of age with a diagnosis of HS were included. Data on comorbidities, HS involvement, and treatment response were collected from medical records. Statistical analysis of the obtained data and a survey of ultrasound images of differential diagnoses were performed. The definition of differential diagnoses was carried out based on a literature review using the terms "mammary" and "fistula".

The study was conducted following the Code of Ethics (Declaration of Helsinki) and all participants provided an Informed Consent Form (ICF) – CAAE: 77223323.1.0000.5404.

Of the 108 patients with HS, 22 showed breast involvement (21 women – 30.8% and one man – 2.5%) with a mean BMI of 34.2, and 18.2% were smokers. Regarding breast involvement, 63.6% had inframammary lesions, 4.5% intermammary lesions, 13.6% had a nodular pattern, and 9.1% a mixed pattern. Two patients (9.09%) had an axillomammary pattern, and two patients showed areolar involvement concomitant with the mixed and axillomammary pattern.

The group of patients with breast involvement had 13 (59%) with Hurley classification III, six patients with Hurley II, and three patients with Hurley I. These same patients had 86% axillary lesions, 82% inguinal lesions, 32% gluteal lesions, and 9% perineal lesions. One patient exclusively showed breast involvement. The presence of breast lesions does not predict more severe disease or a more advanced Hurley stage (p = 0.91 – Chi-Square Test).

Regarding treatment, 21 patients (95%) used sulfamethoxazole + trimethoprim (SMX-TMP), and 50% started using immunobiologicals due to disease severity (seven patients with adalimumab, two with infliximab, and two with secukinumab). When comparing breast lesions that predict a worse response to adalimumab treatment, there is no statistical relationship (p = 0.077 – Chi-square test).

Three (13.6%) of the patients had surgically treated breast lesions, with one patient using concomitant adalimumab (three years after initiation) and SMX-TMP. Two patients underwent deroofing as a surgical approach.

The previous 2021 classification includes nodular/diffuse (nodules distributed throughout the breast quadrants), sternal (intermammary), frictional (inframammary), and mixed (nodular and frictional) involvement (Fig. 1). The observations in this study identify two important forms of involvement – ​​axillomammary (9.09%) and areolar (4.5%; Fig. 2). Axillomammary involvement occurs due to severe lesions in the axilla that extend to the breasts. The areolar form presents with the formation of draining fistulas within the areolar complex. Both forms require multidisciplinary care from a dermatologist with plastic surgery and/or a mastologist for a surgical approach and reconstruction, aiming for less anatomical impact.

**Fig. 1:**
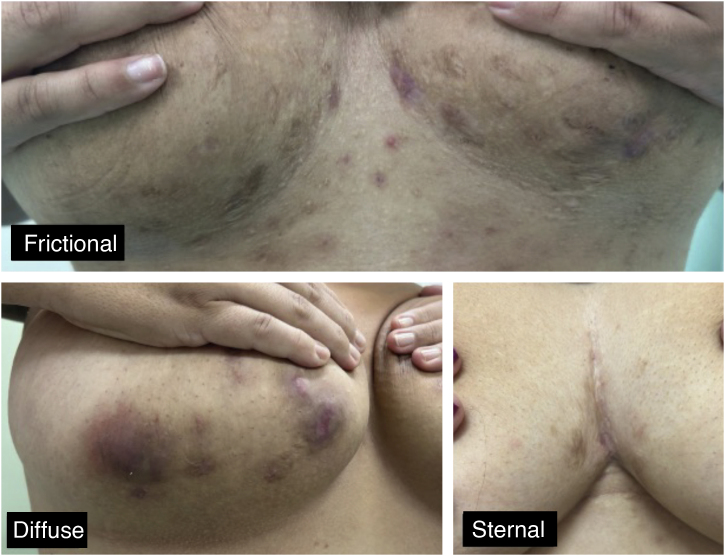
Clinical classification of breast involvement in hidradenitis suppurativa: previously proposed forms with a change from nodular form to diffuse form.

**Fig. 2:**
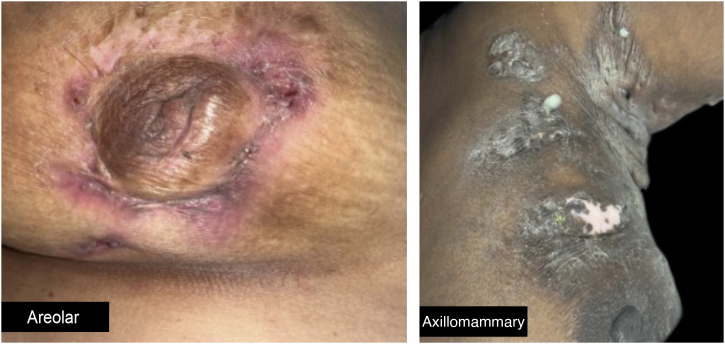
Areolar and axillomammary forms – subtypes of breast involvement by hidradenitis suppurativa to be considered in the classification.

Furthermore, the nodular pattern only shows clinical lesions and not the location of the involvement. The authors propose a change to the term diffuse, characterized by the presence of nodules distributed throughout the breast quadrants, except in the intermammary and/or inframammary region. Thus, all types of breast involvement show the lesion location.

Most patients have more severe forms of HS (59% Hurley III), but breast involvement was not associated with severity, in contrast to a Dutch cohort that showed this association.^5^ In French and Italian cohorts, 28.6% and 16.3% of women and 7.1% and 4.6% of men had breast involvement, but neither had information on the affected sites.^6^, ^7^ The French cohort showed results similar to the present study, possibly due to the similar average BMI, which was considered a risk factor for breast involvement.

The axillomammary pattern has been previously described in a study on cases of HS associated with Behçet's disease,^8^ but the author does not mention other possible breast involvements.

The few excisions of breast lesions highlight the difficulty of surgical treatment. Due to the extensive involvement, there is an indication for wide excisions or even total mastectomies. Areolar involvement is more challenging, as surgical amputation is indicated.

Regarding differential diagnoses, breast neoplasia should be considered if there are many scars or lymphedema, although mammary HS is not associated with malignancy. Other differential diagnoses are squamous metaplasia of the lactiferous ducts, forming abscesses and fistulas in the periareolar region in smoking patients, ^4^ idiopathic granulomatous mastitis, and nipple fistula. Table 1 depicts suggestions for diagnosis and ultrasound findings. The diseases show similar ultrasound findings, as shown in Fig. 3, Fig. 4. These lesions, if accompanied by involvement of other sites, could be diagnosed as HS. Future research should clarify whether such involvements are not part of the same spectrum.

**Table 1:** Differential diagnoses of mammary hidradenitis suppurativa.

**Diagnosis and clinical presentation**	**Ultrasound findings**	**Highlights for diagnosis**
**Mammary HS** ‒ Nodules, abscesses, and fistulas in any location of the breasts.	Thickening and/or altered echogenicity of the dermis, pseudocysts with anechoic or hypoechoic content located in the dermis or hypodermis, fluid collections with anechoic or hypoechoic content connected at the base of hair follicles, fistulous tracts with anechoic or hypoechoic content connected at the base of thickened hair follicles. Increased vascularization on Doppler study.	Involvement of other typical regions such as the armpits and groin. Recurrent nature. Anatomopathological findings include hyperparakeratosis, follicular hyperkeratosis, perifolliculitis, neutrophil accumulation as an abscess, lymphocytic and plasma cell infiltrate, epithelialized sinus tract surrounded by an inflammatory reaction.
**Breast neoplasm** ‒ Painful, fixed, irregular lump in the breasts or axillae, change in skin texture, nipple inversion with discharge ^9^	A nodule with a most commonly irregular shape, with non-circumscribed margins (spiculated/microlobulated/indistinct/angular), heterogeneous, predominantly hypoechoic, which may extend to the skin and nipple-areola complex. It may be vascularized on Doppler.	Isolated lesion, without involvement of the inframammary area. Alteration of the adjacent skin ("peau d'orange"). Confirmation by histopathological examination.
**Squamous metaplasia of the lactiferous ducts (SMOLD)**‒ Periareolar papules, nodules and fistulas, abscesses, nipple retraction. ^4^	Increased echogenicity, predominantly in the periareolar regions, associated with cutaneous thickening in this area. Fluid collections (with anechoic or hypoechoic content, possibly with suspended debris), of varying sizes, which may be associated with fistulous tracts. There may be increased vascularization on Doppler ultrasound.	Isolated involvement of the breasts and areolar region.
**Nipple fistula** ‒ Abscesses and periareolar fistula ^10^	A fistulous tract of the nipple-areola complex, affecting the nipple, with anechoic or hypoechoic contents, and which may extend deep into the breast and/or subcutaneous tissue.	Isolated involvement of the breasts and areolar region.
**Idiopathic granulomatous mastitis** ‒ Erythema and edema of the skin, nodules, nipple retraction, abscesses.	Fluid collections (with anechoic or hypoechoic content, possibly containing suspended debris), which may be associated with a fistulous tract to the skin and nipple-areola complex, in addition to skin thickening or presenting as an irregular nodule. There may be increased vascularization on Doppler.	Histopathological examination with non-caseating granulomas centered on the mammary lobules, history of breastfeeding in the last five years^3^

**Fig. 3:**
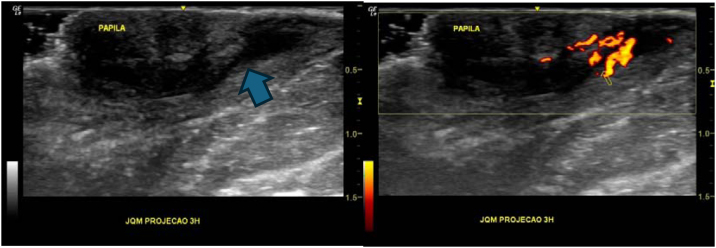
Ultrasound findings: Areolar HS – Fistula in the dermis/hypodermis at the junction of the medial quadrants of the right breast, periareolar, in a 3H projection with communication with a duct in the retroareolar region (blue arrow). Increased locoregional vascularization is noted on Doppler (Power) study.

**Fig. 4:**
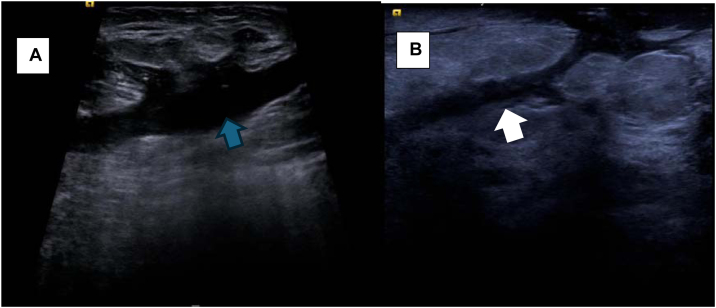
Ultrasound findings of idiopathic granulomatous mastitis – (A) Collection with anechoic content and some suspended debris (blue arrow), located in the inferolateral quadrant of the left breast; (B) Hypoechoic collection, located in the right breast, with a periareolar fistulous tract (white arrow).

The limitations of this study comprise the small sample size and the lack of evaluation of the isolated response of the mammary subtypes. Furthermore, patients come from tertiary centers with more severe symptoms.

Breast involvement in HS is more frequent and diverse than previously recognized and is not necessarily associated with more severe disease. The identification of specific forms, such as axillomammary and areolar forms, reinforces the need to expand the existing classification and directs more individualized therapeutic strategies, often requiring the joint participation of dermatologists, mastologists, and plastic surgeons. The use of the term diffuse instead of nodular, as proposed by the 2021 classification, better defines the location of lesions. The systematic incorporation of ultrasound and careful consideration of differential diagnoses can improve diagnostic accuracy and clinical outcomes. Future investigations should confirm this proposal and evaluate the impact of these subclassifications on clinical decision-making.

## ORCID ID

Carolina M. Stecca: 0009-0009-1259-6212

Thais dos S. Martins: 0009-0001-6965-9239

Luciana V. Gomide: 0009-0001-5465-5462

Juliana Y. Massuda Serrano: 0000-0002-5221-2385

Thais H. Buffo: 0000-0002-6833-7596

Renata F. Magalhães: 0000-0001-9170-932X

## Financial support

None declared.

## Authors' contributions

Ariany T.A.S. Denofre: Design and planning of the study; collection of data, or analysis and interpretation of data; drafting and editing of the manuscript; collection, analysis, and interpretation of data; intellectual participation in the propaedeutic and/or therapeutic conduct of the studied cases; critical review of the literature.

Carolina M. Stecca: Collection, analysis, and interpretation of data; effective participation in research orientation; intellectual participation in the propaedeutic and/or therapeutic conduct of the studied cases.

Thais dos S. Martins: Collection, analysis, and interpretation of data; effective participation in research orientation; intellectual participation in the propaedeutic and/or therapeutic conduct of the studied cases.

Luciana V. Gomide: Collection, analysis, and interpretation of data; effective participation in research orientation; intellectual participation in the propaedeutic and/or therapeutic conduct of the studied cases.

Juliana Y. Massuda Serrano: Collection, analysis, and interpretation of data; effective participation in research orientation; intellectual participation in the propaedeutic and/or therapeutic conduct of the studied cases.

Thais H. Buffo: Collection, analysis, and interpretation of data; effective participation in research orientation; intellectual participation in the propaedeutic and/or therapeutic conduct of the studied cases.

Renata F. Magalhães: Design and planning of the study; critical review of important intellectual content; effective participation in research orientation; intellectual participation in the propaedeutic and/or therapeutic conduct of the studied cases; approval of the final version of the manuscript.

## Research data availability

The entire dataset supporting the results of this study was published in this article.

## Conflicts of interest

None declared.
